# Supplementary material for: Mechanisms and function of de novo DNA methylation in placental development reveals an essential role for DNMT3B
Source: Nat Commun. 2023 Jan 23;14:371. doi: 10.1038/s41467-023-36019-9 (PMC9870994; doi:10.1038/s41467-023-36019-9)
Supplement: Supplementary file 1 — Supplementary Information [file 41467_2023_36019_MOESM1_ESM.pdf]

Supplementary Figures

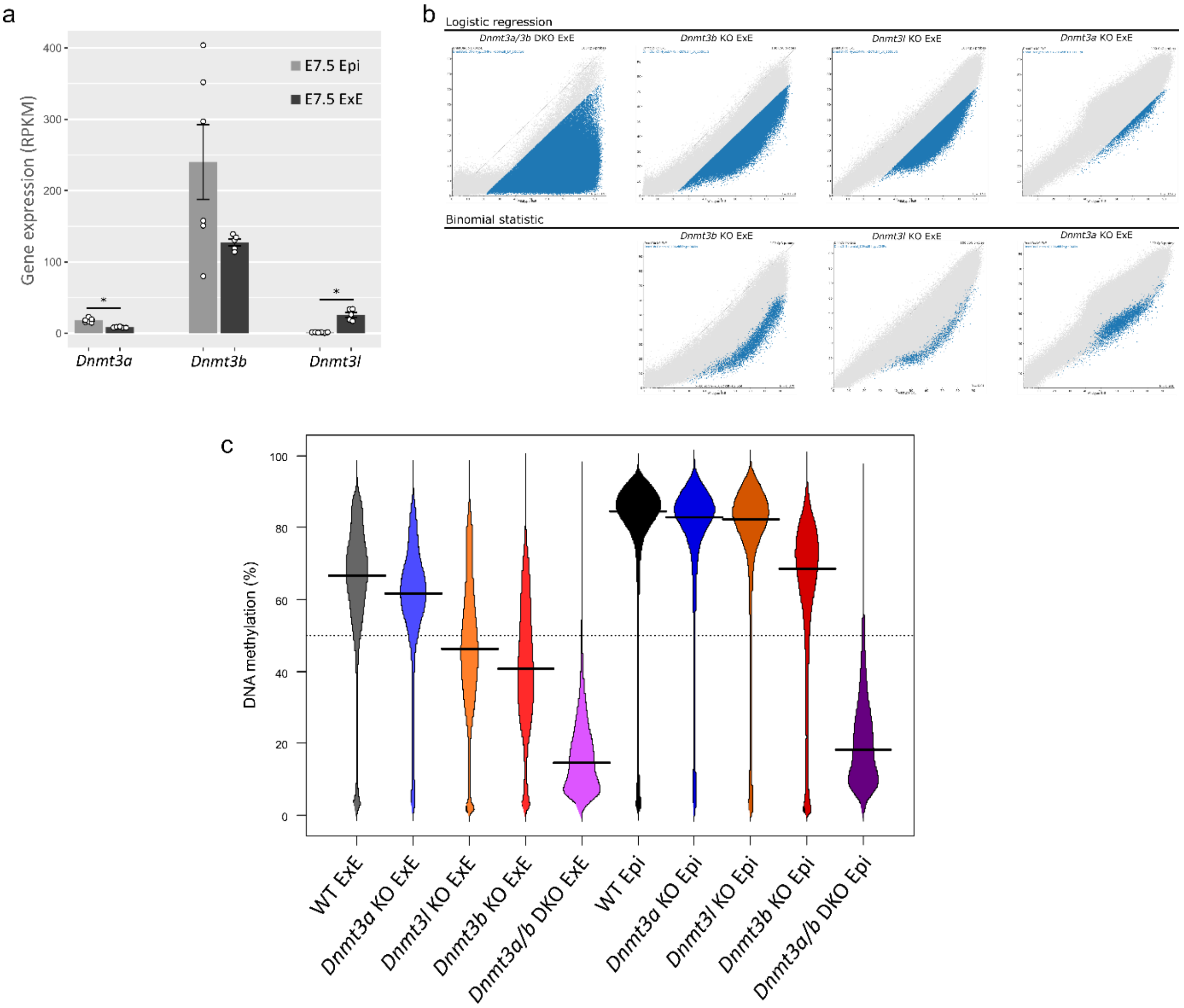

Supplementary Fig 1. a) The barplot shows the expression levels of *Dnmt3a*, *Dnmt3b* and *Dnmt3l* genes in E7.5 epiblast (Epi) (N=6) and extra-embryonic ectoderm (ExE) (N=5) using publicly available RNA-seq data[1]. Gene expression was quantitated as RPKM, merging all isoforms. The error bars show standard error of the mean, asterisks ($p<0.0001$) show significant pair-wise comparisons (two-tailed t-test). **b)** The scatterplots show DNA methylation between KO and WT E7.5 ExE for *Dnmt3a/b* DKO, *Dnmt3b*, *Dnmt3l* and *Dnmt3a* KOs, using autosomal 100-CpG windows with at least 10 informative CpGs per replicate. Significantly hypomethylated differentially methylated regions are shown in blue, defined using logistic regression and a difference >20% (top panel) or a binomial test with a minimum 10% difference (bottom panel). **c)** The beanplot shows the DNA methylation levels of autosomal 100-CpG windows for ExE and epiblast (Epi) from WT, *Dnmt3a/b* DKO, *Dnmt3b* KO, *Dnmt3l* KO and *Dnmt3a* KO E7.5 embryos. The horizontal bars show the median.

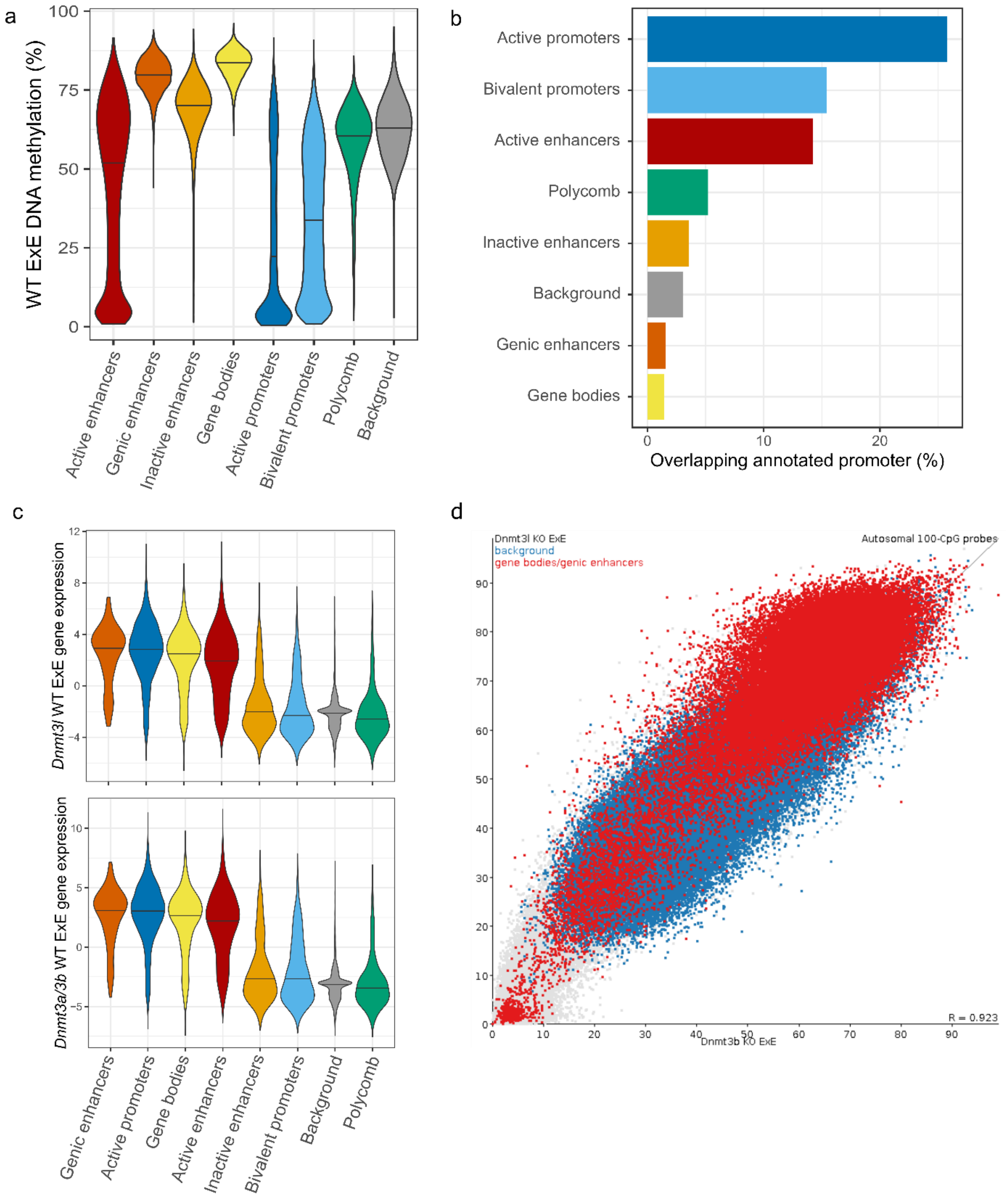

Supplementary Fig 2. a) The beanplot shows DNA methylation levels for each chromatin feature in E7.5 WT ExE. The horizontal bars show the median. **b)** The barplot shows the overlap between annotated gene promoters and chromatin features identified in the UMAP dimensionality reduction and clustering of 100-CpG windows using histone modification enrichment in ExE (Fig. 2b, Methods). **c)** The beanplots show expression levels of genes associated with chromatin features in *Dnmt3a/b* WT ExE (N=5) and *Dnmt3l* WT ExE (N=3). The horizontal bars show the median. **d**) The scatterplot shows DNA methylation between *Dnmt3b* KO and *Dnmt3l* KO E7.5 ExE, using autosomal 100-CpG windows with at least 10 informative CpGs per replicate. Windows that fall within chromatin features are highlighted: background in blue (N=137,069) and gene bodies or genic enhancers in red (N=50,027).

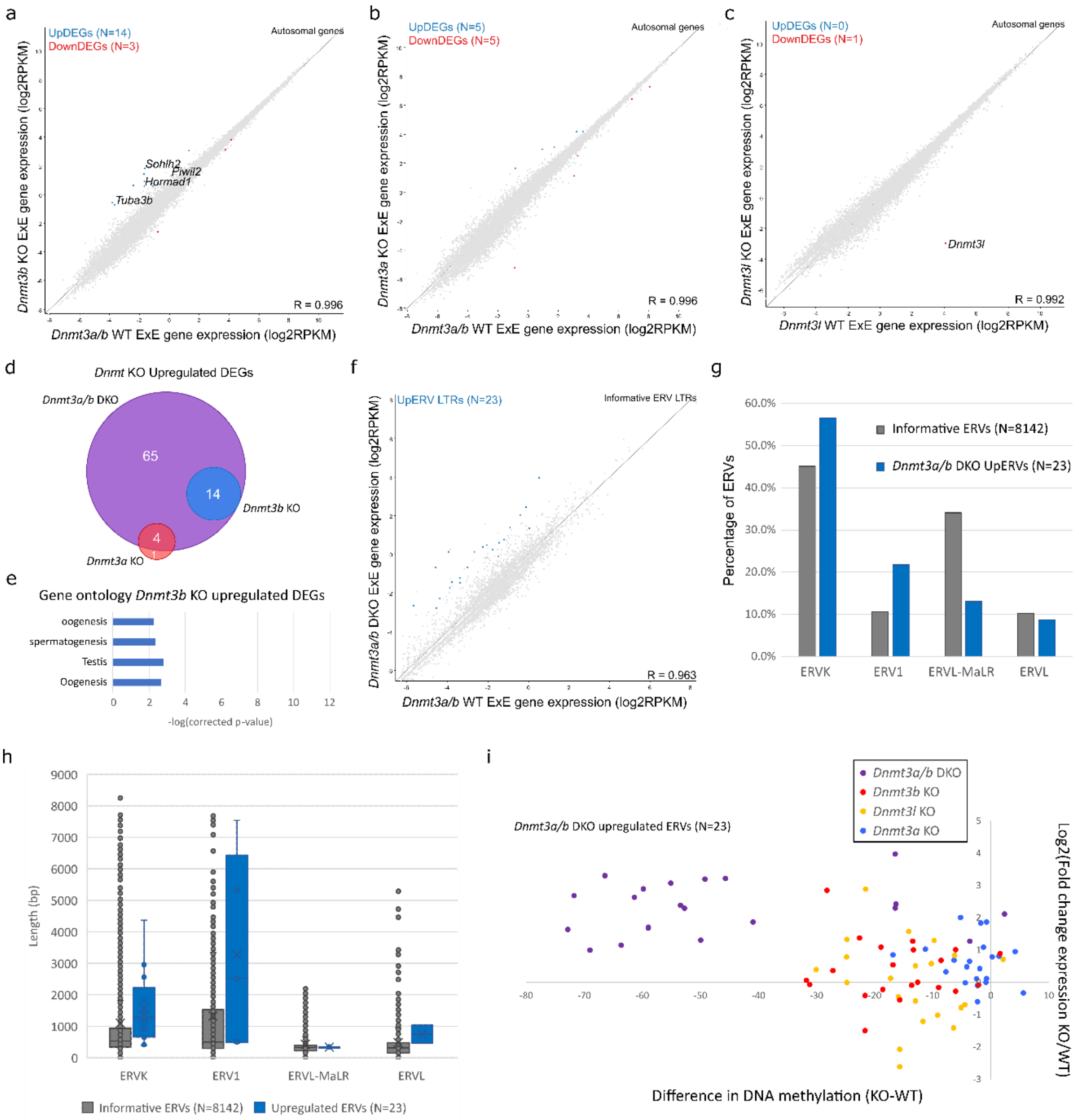

Supplementary Fig. 3 a) The scatterplot compares gene expression between *Dnmt3b* KO and WT control ExE for autosomal genes. Up- and down-regulated differentially expressed genes (DEGs) are highlighted in blue and red, respectively. **b)** The scatterplot compares gene expression between *Dnmt3a* KO and WT control ExE for autosomal genes. Up- and down-regulated differentially expressed genes (DEGs) are highlighted in blue and red, respectively. **c)** The scatterplot compares gene expression between *Dnmt3l* KO and WT control ExE for autosomal genes. Up- and down-regulated differentially expressed genes (DEGs) are highlighted in blue and red, respectively. **d)** The Venn diagram shows the proportion of overlap between up-regulated DEGs identified in the *Dnmt3a/b* DKO (N=83), *Dnmt3a* KO (N=5) and *Dnmt3b* KO ExE (N=14). **e)** The bar plot shows significant gene ontology categories enriched among *Dnmt3b* KO up-regulated genes in E7.5 ExE, using Fisher Exact Test with Benjamini-Hochberg correction for multiple comparisons. **f)** The scatterplot compares expression between *Dnmt3a/b* DKO and WT control ExE for autosomal ERVs with at least one same-stranded read in any replicate and not overlapping an annotated gene (Informative ERV LTRs, N=8,142). Significant up-regulated ERVs (blue) were identified using LIMMA t-test statistic (p<0.05, correcting for multiple comparisons using Benjamini-Hochberg correction). **g)** The barplot shows the distribution of ERV repeat classes among ERVs upregulated in *Dnmt3a/b* DKO and informative ERVs (p=0.09, two-tailed

Chi-square statistic). **h)** The boxplot shows the base-pair (bp) length of ERVs upregulated in *Dnmt3a/b* DKO and informative ERVs. The mean length of ERVs upregulated in *Dnmt3a/b* DKO (N=23) was compared to informative ERVs (N=8142) using a two-tailed t-test with unequal variances (p=0.03). The boxplot centre line is the median and the X is the mean, with box limits showing the upper and lower quartiles, whiskers as 1.5x interquartile range and dots as outliers. **i)** The scatterplot shows the change in DNA methylation (KO-WT) of 100-CpG windows overlapping ERVs up-regulated in the *Dnmt3a/b* DKO (N=23) with log2 fold-change in ERV expression (RPKM+0.1) quantified in the *Dnmt3/3b* DKO, *Dnmt3b* KO, *Dnmt3a* KO and *Dnmt3l* KO.

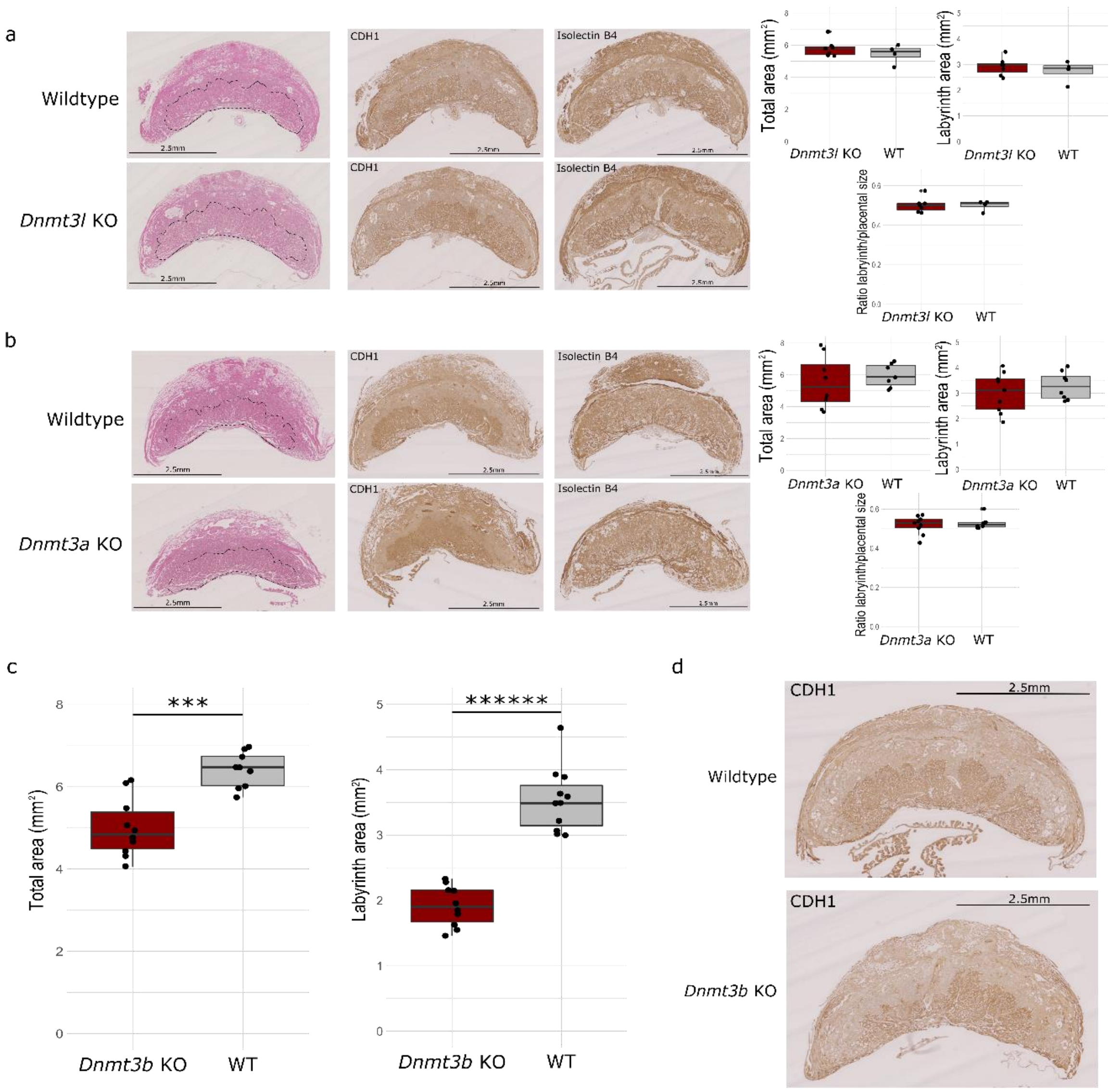

Supplementary Fig. 4 a) Representative haematoxylin and eosin staining (left), E-cadherin (CDH1) immunohistochemistry (middle) and isolectin BSI-B4 immunohistochemistry (right) of E12.5 placentas from a *Dnmt3l* KO and littermate WT control. The boxplots show the total placenta area (top left), labyrinth area (top right), and the ratio of labyrinth to placental size (bottom) in *Dnmt3l* KO (N=7) and WT (N=4) E12.5 placentas. Pairwise comparisons were done by two-tailed t-test (not significant). For all boxplots shown in this figure, boxplot centre line is the median, with box limits showing the upper and lower quartiles and whiskers as 1.5x interquartile range. Individual data points are shown as dots. **b)** Representative haematoxylin and eosin staining (left), E-cadherin (CDH1) immunohistochemistry (middle) and isolectin BSI-B4 immunohistochemistry (right) of E12.5 placentas from a *Dnmt3a* KO and littermate WT control. The boxplots show the total placenta area (top left), labyrinth area (top right), and the ratio of labyrinth to placental size (bottom) in *Dnmt3a* KO (N=9) and WT (N=8) E12.5 placentas. Pairwise comparisons were done by two-tailed t-test (not significant). **c)** The boxplots show the total placenta area (left), labyrinth area (right), in *Dnmt3b* KO (N=11) and WT (N=10) E12.5 placentas. Pairwise comparisons were done by two-tailed t-test (***p=0.00009, ******p=0.00000003). **d)** Representative E-cadherin (CDH1) immunohistochemistry of E12.5 placentas from a *Dnmt3b* KO and littermate WT control.

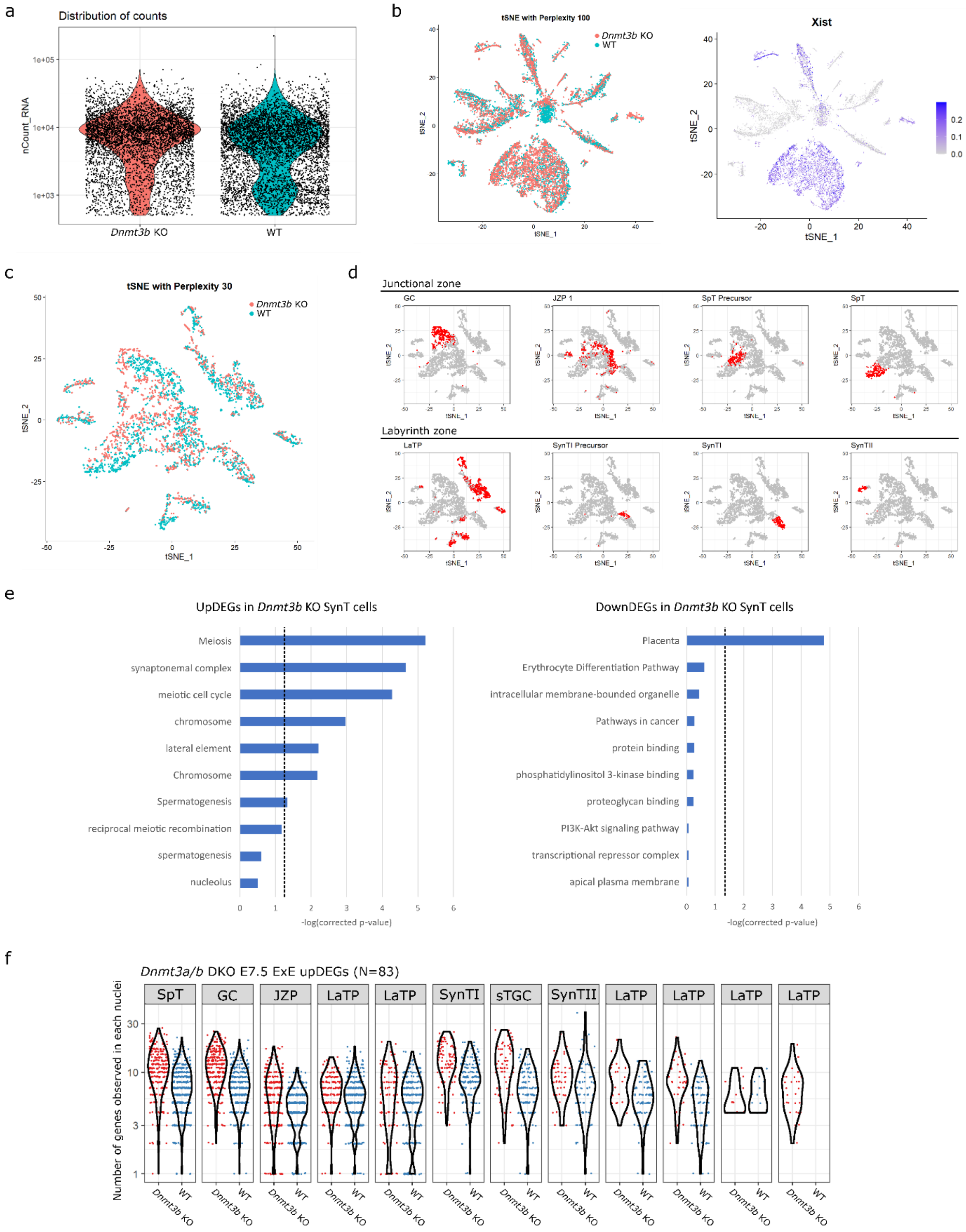

Supplementary Fig. 5 a) The beanplot shows the matched distributions of read counts per cell in single-nuclei RNA-seq data from *Dnmt3b* KO and WT E12.5 placentas. **b)** The tSNE plot shows the clustering of single nuclei in *Dnmt3b* KO and WT E12.5 placentas (left). Nuclei with high levels of *Xist* expression (right) are identified as maternal cells, and were excluded from subsequent analysis. **c)** The *Dnmt3b* KO and WT single-nuclei RNA-seq data were then re-clustered after maternal cells were excluded, as shown in the tSNE plot. **d)** Using published reference lists to identify distinct cell types within E12.5 placentas[2], we classified cell types within our data (shown in red) from the junctional zone (top) and labyrinth zone (bottom). Acronyms: GC=glycogen cell; JZP=junction zone trophoblast;

SpT=spongiotrophoblast; LaTP=labyrinth trophoblast; SynT=syncytiotrophoblast. **e)** The bar plot shows the top 10 gene ontology categories identified among *Dnmt3b* KO up- and down-regulated differentially expressed genes (DEGs) in SynTI and SynTII cells, using Fisher Exact Test with Benjamini-Hochberg correction for multiple comparisons. The dashed line shows the corrected p-value<0.05 significance threshold. **f)** The beanplots show the number of *Dnmt3a/b* E7.5 ExE upregulated DEGs (N=83) detected in each cell, within each cell type cluster identified in E12.5 placentas between *Dnmt3b* KO and WT.

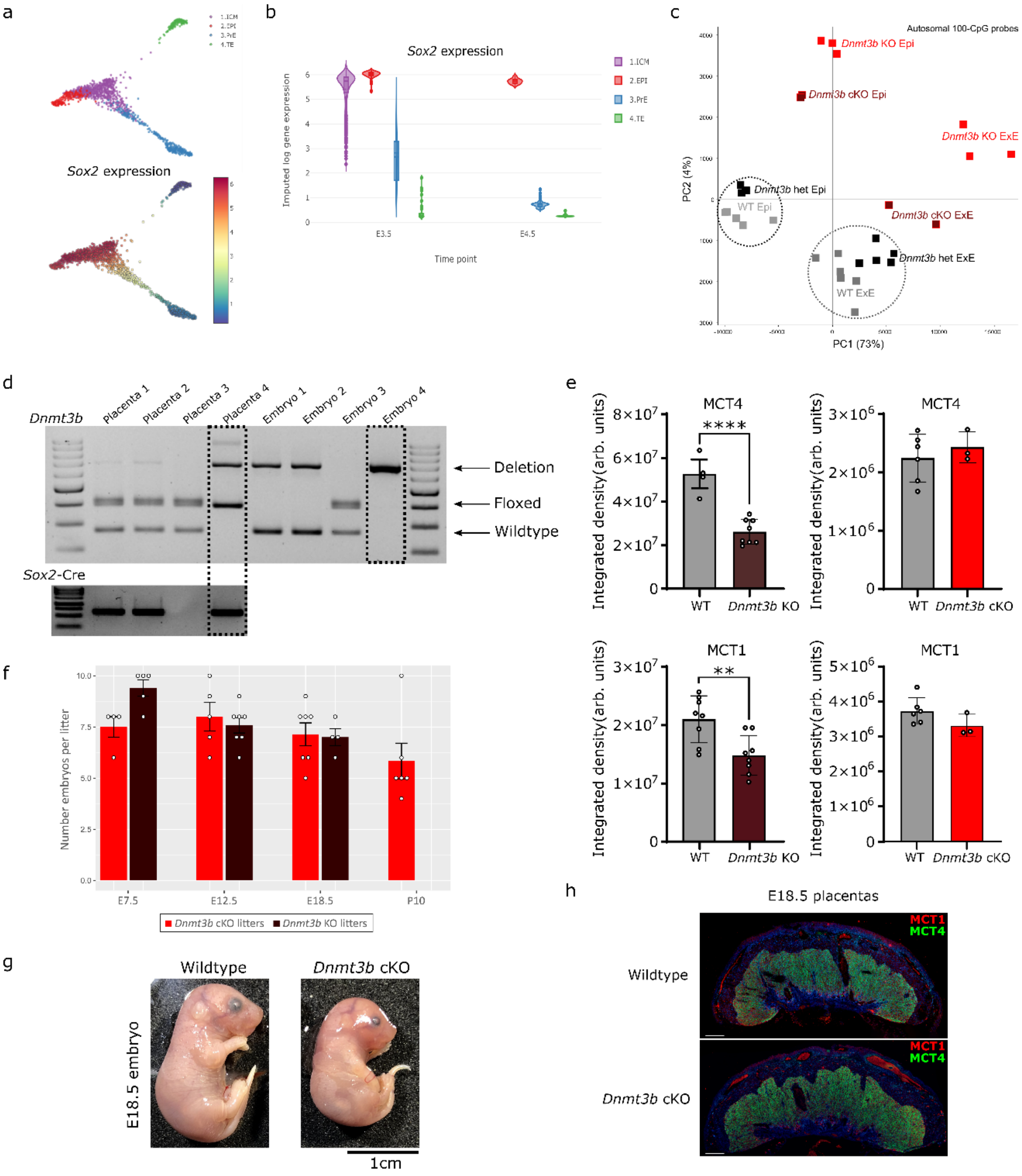

Supplementary Fig. 6 a) The tSNE plot shows single cells from inner cell mass (ICM), epiblast (EPI), primitive endoderm (PrE), and trophectoderm (TE) in E3.5 and E4.5 embryos (top) and levels of *Sox2* expression (bottom)[3]. **b)** The beanplot shows the levels of imputed gene expression for *Sox2* among inner cell mass (ICM), epiblast (EPI), primitive endoderm (PrE), and trophectoderm (TE) cells in E3.5 and E4.5 embryos[3]. The boxplot centre line is the median and the dashed line is the mean, with box limits showing the upper and lower quartiles, whiskers as 1.5x interquartile range and dots as outliers. **c)** The PCA plot shows DNA methylation replicates for E7.5 Epi and ExE from *Dnmt3b* WT, *Dnmt3b* heterozygotes (het), *Dnmt3b* cKO using Sox2-Cre, and *Dnmt3b* KOs. *Dnmt3b* cKO Epi cluster near *Dnmt3b* KO Epi, reflecting their highly correlated global DNA methylation patterns (R=0.957) and supports that there is a similar loss of DNMT3B activity in these KOs. DNA methylation was quantitated for autosomal 100-CpG windows with at least 10 informative CpGs. **d)** The agarose gel image shows the genotyping results for *Dnmt3b* allele (top) and the *Sox2*-Cre (bottom) from E8.5 whole embryo and placentas collected from a set of embryos from a *Dnmt3b* homozygous floxed (fl/fl) female bred

with a *Dnmt3b* -/+, Sox2-Cre positive male. Embryo 4 is a *Dnmt3b* cKO, showing the presence of only the deletion allele in the embryo, while retaining the intact floxed allele in the placenta. A 100bp ladder (GeneRuler), with the lowest shown band=200bp, is the marker on the top gel image; while a 1kb ladder (GeneRuler) is the marker on the bottom gel image. The approximate expected fragment sizes for *Dnmt3b* wildtype allele=270bp, floxed allele=400bp, deletion allele=700bp and Cre=650bp. **e)** The barplots show the mean quantification of MCT4 (top) and MCT1 (bottom) for *Dnmt3b* KO (left) and *Dnmt3b* cKO (right) compared to littermate wildtype (WT) controls, measured as integrated density (arb. units = arbitrary units). Pairwise comparisons were done by two-tailed t-test (**p=0.005, ****p=5.7E-7). Individual data points are shown as dots and error bars show standard deviation. **f)** The barplot shows the mean number of embryos per litter assessed at E7.5, E12.5, E18.5 and P10 to evaluate the frequency of *Dnmt3b* cKO (N=4, 5, 7 and 6 litters, respectively) and *Dnmt3b* KO (N=5, 7, 4 and 0 litters, respectively) embryos (shown in Fig. 5e). As there were no *Dnmt3b* KO embryos at E18.5, these were not evaluated at P10. *Dnmt3b* cKO pairs comprise a *Dnmt3b* fl/fl or fl/+ female bred with a *Dnmt3b* -/+, Sox2-Cre positive male, while *Dnmt3b* KO pairs comprise a Dnmt3b -/+ female and male. There were no significant differences in litter size between *Dnmt3b* cKO and *Dnmt3b* KO at E7.5, E12.5 or E18.5, using pairwise two-tailed t-test comparisons with Bonferroni-corrected p-value threshold for multiple comparisons. The error bars show standard error of the mean. **g)** Images show E18.5 embryos from *Dnmt3b* cKO and WT littermate control. **h)** Immunofluorescence for MCT1 and MCT4, which stain for syncytiotrophoblast layer I (SynTI) and SynTII that separate maternal and foetal circulation, respectively, of E18.5 placentas from a *Dnmt3b* KO and littermate WT control. White scale bar = 100µm.

Supplementary references

1. Hanna, C. W. *et al*. Endogenous retroviral insertions drive non-canonical imprinting in extra-embryonic tissues. *Genome Biol.* **20**, 225-019-1833-x (2019).

2. Marsh, B. & Blelloch, R. Single nuclei RNA-seq of mouse placental labyrinth development. *Elife* **9**, 10.7554/eLife.60266 (2020).

3. Nowotschin, S. *et al*. The emergent landscape of the mouse gut endoderm at single-cell resolution. *Nature* **569**, 361-367 (2019).
