# Supplementary material for: Mechanisms and function of de novo DNA methylation in placental development reveals an essential role for DNMT3B
Source: Nat Commun. 2023 Jan 23;14:371. doi: 10.1038/s41467-023-36019-9 (PMC9870994; doi:10.1038/s41467-023-36019-9)
Supplement: Supplementary file 3 — Description of Additional Supplementary Files [file 41467_2023_36019_MOESM3_ESM.pdf]

Description of Additional Supplementary Files

File Name: Supplementary Data 1
Description: Datasets generated for this study.

File Name: Supplementary Data 2
Description: Statistical comparison of enrichment for specific chromatin features among differentially methylated domains identified in ExE in Dnmt3a, Dnmt3b, and Dnmt3l KOs (identified by binomial statistic). Enrichment or depletion of the most hypomethylated DMRs at chromatin features in Dnmt3a, Dnmt3b and Dnmt3l KOs were statistically compared to a random set of 100-CpG windows using one-proportion Z test with Yates' continuity correction.

File Name: Supplementary Data 3
Description: Differentially expressed genes identified in Dnmt3a/b DKO E7.5 ExE compared to WT, using DESeq2 with Benjamini-Hochberg correction for multiple testing and a minimum 1.5- fold-change in expression.

File Name: Supplementary Data 4
Description: Differentially expressed genes identified in Dnmt3b KO E12.5 SynT cells, identified using DESeq2 with Benjamini-Hochberg correction for multiple testing.
