# Supplementary material for: Mechanisms and function of de novo DNA methylation in placental development reveals an essential role for DNMT3B
Source: Nat Commun. 2023 Jan 23;14:371. doi: 10.1038/s41467-023-36019-9 (PMC9870994; doi:10.1038/s41467-023-36019-9)
Supplement: Supplementary file 5 — Reporting Summary [file 41467_2023_36019_MOESM5_ESM.pdf]

nature portfolio

Corresponding author(s): Courtney Hanna

Last updated by author(s): 14 Dec 2022

Reporting Summary

Nature Portfolio wishes to improve the reproducibility of the work that we publish. This form provides structure for consistency and transparency in reporting. For further information on Nature Portfolio policies, see our Editorial Policies and the Editorial Policy Checklist.

Statistics

For all statistical analyses, confirm that the following items are present in the figure legend, table legend, main text, or Methods section.

n/a	Confirmed	
☐	☒	The exact sample size (*n*) for each experimental group/condition, given as a discrete number and unit of measurement
☐	☒	A statement on whether measurements were taken from distinct samples or whether the same sample was measured repeatedly
☐	☒	The statistical test(s) used AND whether they are one- or two-sided<br>*Only common tests should be described solely by name; describe more complex techniques in the Methods section.*
☒	☐	A description of all covariates tested
☐	☒	A description of any assumptions or corrections, such as tests of normality and adjustment for multiple comparisons
☐	☒	A full description of the statistical parameters including central tendency (e.g. means) or other basic estimates (e.g. regression coefficient) AND variation (e.g. standard deviation) or associated estimates of uncertainty (e.g. confidence intervals)
☐	☒	For null hypothesis testing, the test statistic (e.g. *F*, *t*, *r*) with confidence intervals, effect sizes, degrees of freedom and *P* value noted<br>*Give P values as exact values whenever suitable.*
☒	☐	For Bayesian analysis, information on the choice of priors and Markov chain Monte Carlo settings
☐	☒	For hierarchical and complex designs, identification of the appropriate level for tests and full reporting of outcomes
☐	☒	Estimates of effect sizes (e.g. Cohen's *d*, Pearson's *r*), indicating how they were calculated

Our web collection on statistics for biologists contains articles on many of the points above.

Software and code

Policy information about availability of computer code

Data collection	Primary sequencing data was collected using Illumina Next Generation Sequencing Platforms. Imaging data was collected using Hamamatsu NDP.View2 2.9.25 software and Leica Aperio ImageScope software Version 12.3.3.5048 .
Data analysis	Publically available software: Bowtie v2.2.9, Trim Galore v0.6.6, HISAT2 v2.1.0, Bismark v0.16.3, R Studio v1.1.456, R v4.2.0, SeqMonk v1.48.0, CellRanger v6.0.0, https://endoderm-explorer.com/, DAVID Bioinformatics Resource, Excel, Vassarstats.net, R package ggplot2, and GraphPad Prism 9.

For manuscripts utilizing custom algorithms or software that are central to the research but not yet described in published literature, software must be made available to editors and reviewers. We strongly encourage code deposition in a community repository (e.g. GitHub). See the Nature Portfolio guidelines for submitting code & software for further information.

Data

Policy information about availability of data

All manuscripts must include a data availability statement. This statement should provide the following information, where applicable:

- Accession codes, unique identifiers, or web links for publicly available datasets
- A description of any restrictions on data availability
- For clinical datasets or third party data, please ensure that the statement adheres to our policy

Sequencing data generated for this study has been deposited in Gene Expression Omnibus (GEO) under accession number GSE203462.

Field-specific reporting

Please select the one below that is the best fit for your research. If you are not sure, read the appropriate sections before making your selection.

☒ Life sciences ☐ Behavioural & social sciences ☐ Ecological, evolutionary & environmental sciences

For a reference copy of the document with all sections, see nature.com/documents/nr-reporting-summary-flat.pdf

Life sciences study design

All studies must disclose on these points even when the disclosure is negative.

Sample size	For PBAT (bisulphite-seq) experiments, 3 biological replicates per group provide optimal statistical power to detect biologically meaningful differences. This was determined using a power calculation (G*Power) to evaluate DNA methylation changes of >20% between groups, with a 5% standard deviation, a significance threshold of p<0.05 and power of 90%. For RNA-seq, 3-4 biological replicates per group were used to detect a 1.5-fold change in expression, with an expected 0.1-0.2 standard deviation, a significance threshold of p<0.05 and a power of 80%. Two replicates were used for ultra-low input ChIP-seq experiments, as these data were not used for pairwise comparisons and this is consistent with the standard in the field. For phenotyping experiments, a minimum of 4 biological replicates per group were analysed from at least two independent litters, providing us with the statistical power to detect a large effect size, with a significance threshold of p<0.05 and a power of 80%.
Data exclusions	One ultra-low input ChIP-seq dataset for H3K27ac was excluded due to poor signal-to-noise, as evaluated by cumulative distribution plot, and a poor correlation coefficient between replicates (r=0.66); an additional replicate was generated to replace this poor library. Four replicates of ultra-low input RNA-seq libraries were excluded due to poor amplification and consequently high duplication (>70%).
Replication	Reproducibility of sequencing data was evaluated by hierarchical clustering (correlation coefficients) and principle component analysis (PCA), which are presented in the manuscript. replicates for sequencing experiments included replicates from at least two independent litters per knockout model. For phenotyping analyses, placental size, labyrinth size/proportion, and foetal weights were statistical compared between groups using pairwise statistical methods (reported in detail in the manuscript). Placental measurements were assessed among embryos from at least two independent litters (range: 2-4 litters) per knockout model, while foetal weights in the Dnmt3b cKO were assessed across 4 litters at each time point. All immunostaining experiments were performed at least twice for each sample in two independent experiments.
Randomization	For both sequencing experiments and immunostaining/immunohistochemistry, knockout (KO) embryos were processed in parallel with WT controls, and wherever possible, these were matched littermate WT controls. Samples were allocated into groups based on genotypes. No co-variates, other than litter-matching, were controlled for.
Blinding	Investigators were blinded during sample collections, as all genotyping was done posthoc. Phenotypes of placentas were assessed by two independent investigators using hemotoxalyin and eosin stained sections for each knockout strain; genotypes were blinded to assessors.

Reporting for specific materials, systems and methods

We require information from authors about some types of materials, experimental systems and methods used in many studies. Here, indicate whether each material, system or method listed is relevant to your study. If you are not sure if a list item applies to your research, read the appropriate section before selecting a response.

Materials & experimental systems

n/a	Involved in the study	
☐	☒	Antibodies
☒	☐	Eukaryotic cell lines
☒	☐	Palaeontology and archaeology
☐	☒	Animals and other organisms
☒	☐	Human research participants
☒	☐	Clinical data
☒	☐	Dual use research of concern

Methods

n/a	Involved in the study	
☐	☒	ChIP-seq
☒	☐	Flow cytometry
☒	☐	MRI-based neuroimaging

Antibodies

Antibodies used	Anti-H3K4me1 (Active Motif, 39298), Anti-H3K27ac (Abcam, ab4729), Anti Cdh1: anti-E-Cadherin (CDH1) (BD Biosciences, 610181), Biotin-conjugated isolectin from Bandeiraea simplicifolia BSI-B4 (Sigma, L2140), Anti-MCT4 (Merck Millipore, AB3314P), Anti- MCT1 (Merck Millipore, AB1286I), Anti-Mouse IgG (H + L)-HRP (goat polyclonal) Cat. 170–6516 Bio-Rad, Alexa594 (Goat anti-chicken) Invitrogen Cat. A110442, Alexa488 (Donkey anti-rabbit) Invitrogen Cat. A21206, Horseradish peroxidase-conjugated Streptavidin, Vector Laboratories Cat. SA5004
Validation	Active Motif, 39298: The modENCODE and NIH Roadmap Epigenomics Mapping Consortiums have implemented rigorous standardization criteria for all assays and reagents to be used. As part of this initiative, antibody specificity testing and the ability of

the antibodies to work in ChIP-Seq were assessed in a large-scale study. https://www.activemotif.com/catalog/details/39297/histone-h3-monomethyl-lys4-antibody-pab

Abcam, ab4729: Abpromise guarantee covers the use of ab4729 for ChIP-seq experiments. All batches of ab4729 are tested in Peptide Array against peptides to different Histone H3 modifications. Six dilutions of each peptide are printed on to the Peptide Array in triplicate. Results show strong binding to Histone H3 - acetyl K27 peptide (ab24404), indicating that this antibody specifically recognises the Histone H3 - acetyl K27 modification. This antibody has been utilised in 1539 publications. https://www.abcam.com/histone-h3-acetyl-k27-antibody-chip-grade-ab4729.html

Animals and other organisms

Policy information about studies involving animals; ARRIVE guidelines recommended for reporting animal research

Laboratory animals

Several transgenic mouse models were used for this study, maintained on a C57BL6/Babr background. Additionally, adult male and female wildtype C57BL6/Babr and CAST animals were used to generate hybrid embryos. Both male and female embryos were used for this study at embryonic days (E)6.5, E7.5, E8.5, E12.5 and E18.5.

Mice were bred and maintained in the Babraham Institute Biological Support Unit. Since the opening of this barrier facility (2009), no primary pathogens or additional agents listed in the FELASA recommendations (Mähler et al., 2014) have been confirmed during health monitoring surveys of the stock holding rooms. Ambient temperature was approx. 19-21°C and relative humidity approx. 52%. Lighting was provided on a 12 hour light: 12 hour dark cycle including 15 min ‘dawn’ and ‘dusk’ periods of subdued lighting. After weaning, mice were transferred to individually ventilated cages with 1 – 5 mice per cage. Mice were fed CRM (P) VP diet (Special Diet Services) ad libitum and may receive seeds (e.g., sunflower, millet) at the time of cage-cleaning as part of their environmental enrichment. All mouse experimentation was approved by the Babraham Institute Animal Welfare and Ethical Review Body. Animal husbandry and experimentation complied with existing European Union and United Kingdom Home Office legislation and local standards.
Mähler M. et al. FELASA recommendations for the health monitoring of mouse, rat, hamster, guinea pig and rabbit colonies in breeding and experimental units. Laboratory Animals (2014) 48(3): 178-192, DOI:10.1177/0023677213516312

Wild animals

This study did not involve the use of wild animals.

Field-collected samples

This study did not involve the use of samples collected in the field.

Ethics oversight

The use of animals in this study was performed in accordance with the European regulation in Animals (Scientific Procedures) Act 1986 with all protocols approved by the Animal Welfare and Ethical Review Body at the Babraham Institute under licenses issued by the Home Office (UK).

Note that full information on the approval of the study protocol must also be provided in the manuscript.

ChIP-seq

Data deposition

- [x] Confirm that both raw and final processed data have been deposited in a public database such as GEO.
- [ ] Confirm that you have deposited or provided access to graph files (e.g. BED files) for the called peaks.

Data access links
May remain private before publication.

To review GEO accession GSE203462:
Go to https://eur03.safelinks.protection.outlook.com/?url=https%3A%2F%2Fwww.ncbi.nlm.nih.gov%2Fgeo%2Fquery%2Facc.cgi%3Facc%3DGSE203462&data=05%7C01%7Ccwh36%40universityofcambridgecloud.onmicrosoft.com%7C6e2c8538ffe04afa834b08da3c9d20c4%7C49a50445bdfa4b79ade3547b4f3986e9%7C0%7C0%7C637888942824216992%7CUnknown%7CTWFpbGZsb3d8eyJWIjoiMC4wLjAwMDAiLCJQIjoiV2luMzIiLCJBTiI6Ik1haWwiLCJXVCI6Mn0%3D%7C3000%7C%7C%7C&sdata=8jtRtlbgYTIZ2px%2BEX9V7VcdByAG0vuTq%2BCbquDNxK0%3D&reserved=0
Enter token kxadicyslnurlch into the box

Files in database submission

H3K4me1 and H3K27ac libraries were generated for this study, while H3K4me3, H3K27me3 and H3K36me3 libraries were from a published study (Hanna et al. 2019 Genome Biol; 20). Data accessibility information is included in the manuscript.
lane6645_CGATGTTT_B6_CAST_E6_5ExE_H3K27ac_1_L001_R1.fastq.gz
lane6645_CGATGTTT_B6_CAST_E6_5ExE_H3K27ac_1_L001_R3.fastq.gz
lane7736_ACAGTGGT_B6_CAST_E6_5_ExE_H3K4me1_2_L001_R1.fastq.gz
lane7736_ACAGTGGT_B6_CAST_E6_5_ExE_H3K4me1_2_L001_R3.fastq.gz
lane7736_CGATGTTT_B6_CAST_E6_5_ExE_H3K4me1_1_L001_R1.fastq.gz
lane7736_CGATGTTT_B6_CAST_E6_5_ExE_H3K4me1_1_L001_R3.fastq.gz
lane7736_TTAGGCAT_B6_CAST_E6_5_ExE_H3K27ac_3_L001_R1.fastq.gz
lane7736_TTAGGCAT_B6_CAST_E6_5_ExE_H3K27ac_3_L001_R3.fastq.gz
ChIP_replicates_Autosomal_100CpG_windows_RPKM.txt

Genome browser session
(e.g. UCSC)

https://genome.ucsc.edu/s/channa/mm10_ChIPseq_ExE

Methodology

Replicates

Two replicates of each H3K27ac and H3K4me1 ChIP-seq libraries were generated to analyze with previously generated datasets for H3K4me3, H3K27me3, and H3K36me3 (Hanna et al. 2019 Genome Biol; 20), using identical methodology and sample preparation.

Sequencing depth	The average number of raw reads was 25,593,877 (range: 20,057,589-34,312,899) and uniquely mapped reads was 13,466,135 (range: 12,250,084-14,857,283). Libraries were sequenced 75-bp paired-end.
Antibodies	Anti-H3K4me1 (Active Motif, 39298) or Anti-H3K27ac (Abcam, ab4729)
Peak calling parameters	ChIP-seq enrichment was used as a continuous variable in this study, hence peak calling was not performed.
Data quality	Using 1kb running windows, the correlation coefficients for ultra-low input ChIP-seq replicates generated from E6.5 extra-embryonic ectoderm for H3K27ac were r=0.86 and for H3K4me1 were r=0.89. These are comparable to the published ultra-low input ChIP-seq datasets for the same tissue, including H3K4me3 (r=0.99), H3K27me3 (r=0.88) and H3K36me3 (r=0.86) (Hanna et al. 2019 Genome Biol; 20) and published datasets from mouse ES cells (Zhang et al. 2016 Nature; 537).
Software	SeqMonk v1.48.0
