# Supplementary material for: Mechanisms and function of de novo DNA methylation in placental development reveals an essential role for DNMT3B
Source: Nat Commun. 2023 Jan 23;14:371. doi: 10.1038/s41467-023-36019-9 (PMC9870994; doi:10.1038/s41467-023-36019-9)
Supplement: Supplementary file 6 — Source Data [file 41467_2023_36019_MOESM6_ESM.zip › Source data file_2.pptx]

## Slide 1
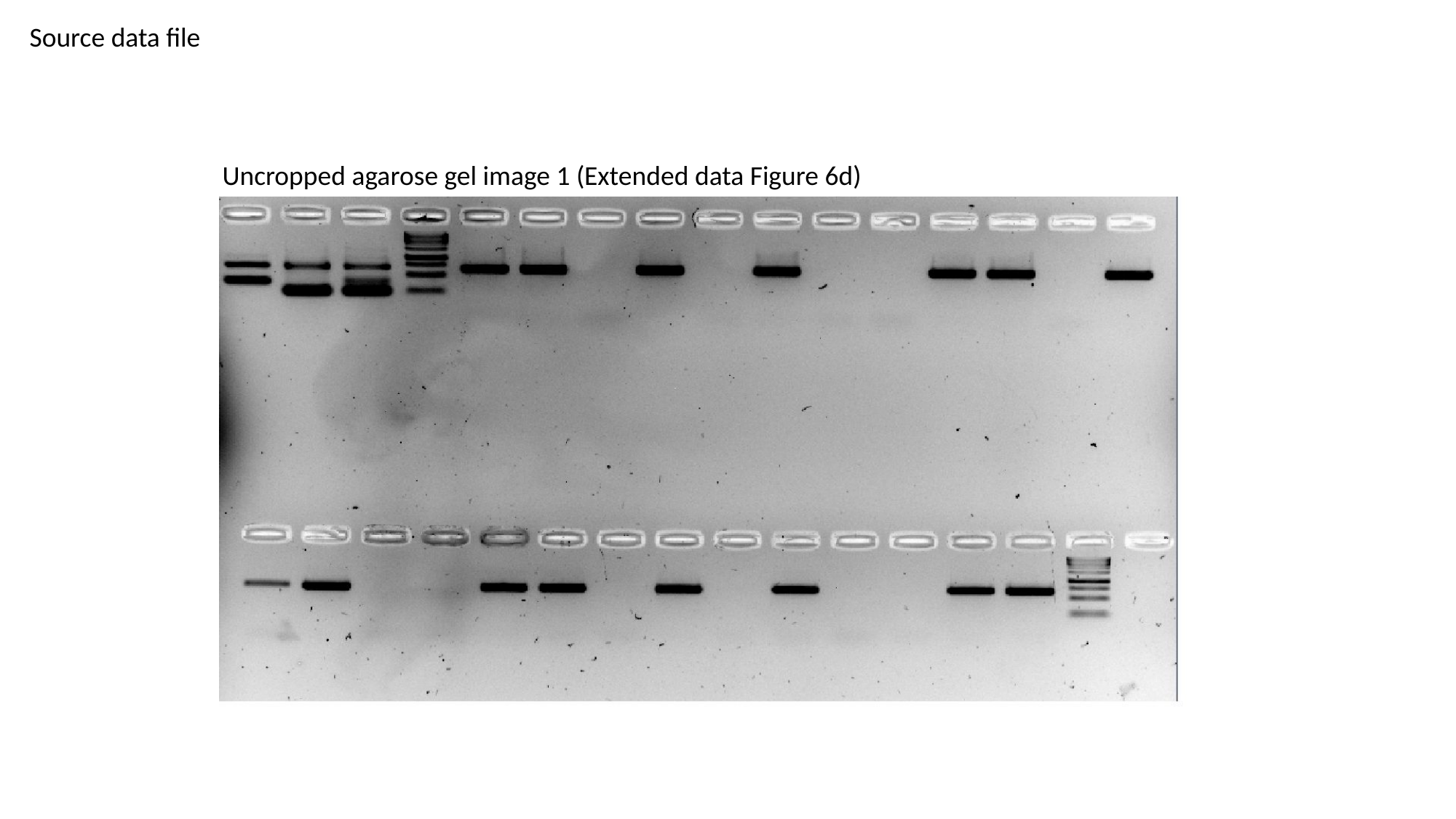

Source data file
Uncropped agarose gel image 1 (Extended data Figure 6d)

## Slide 2
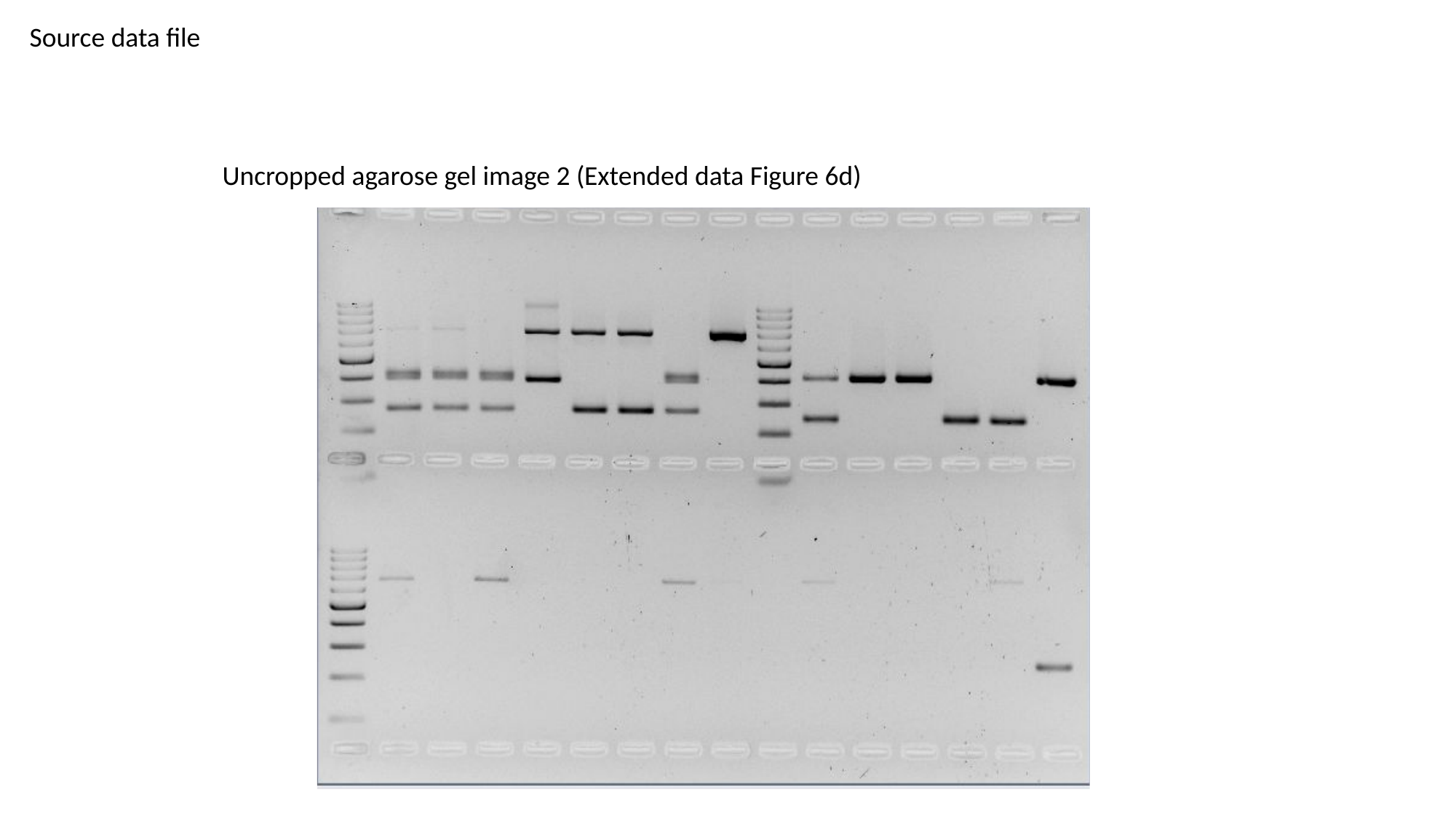

Source data file
Uncropped agarose gel image 2 (Extended data Figure 6d)
